# Mesenchymal Stem Cells Reduce Inflammation in a Mouse Model of Lyme Arthritis

**DOI:** 10.1155/sci/4363386

**Published:** 2025-07-28

**Authors:** Weijiang Ma, Jing Kong, Mengqin Zhang, Hanxin Wu, Shanshan Wan, Xin Liu, Bingxue Li, Yan Dong, Lei Zhong, Weijie Ma, Li Gao, Xinya Wu, Li Peng, Suyi Luo, Zhenhua Ji, Yuxin Fan, Jingjing Chen, Meixiao Liu, Liangyu Zhu, Xun Huang, Rui Yang, Jieqin Song, Fukai Bao, Aihua Liu

**Affiliations:** ^1^Faculty of Basic Medical Sciences, Kunming Medical University, Kunming 650500, Yunnan, China; ^2^Department of Thoracic Surgery, The Second Affiliated Hospital of Kunming Medical University, Kunming 650101, Yunnan, China; ^3^Department of Radiology, The Second Affiliated Hospital of Kunming Medical University, Kunming 650101, Yunnan, China; ^4^Yunnan Health Cell Biotechnology LTD, Kunming 650031, China

**Keywords:** *Borrelia burgdorferi*, Kunming mice, lyme arthritis, lyme disease, mesenchymal stem cells

## Abstract

Lyme disease (LD), a zoonotic infectious disease caused by *Borrelia burgdorferi* (*B. burgdorferi*), can affect various organs, including the skin, heart, nervous system, and joints. Lyme arthritis (LA) is the most common and severe late-stage presentation of LD, often presenting with intermittent joint swelling and pain. Although antibiotics are effective in most patients with LA, some patients may continue to experience arthritis symptoms for months or years after standard treatment, which poses a serious threat to their quality of life. Therefore, more effective treatments are urgently needed. The purpose of this study was to evaluate the therapeutic effects of human umbilical cord mesenchymal stem cells (hUC-MSCs) on LD in Kunming (KM) mice. A bilateral hind limb LA model was established by infecting KM mice with *B. burgdorferi*. Low and high doses of hUC-MSCs (1 × 10^6^ and 2 × 10^6^ cells, respectively) were injected (one time every 2 days) into the right tibiotalar joints of the mice, whereas the left tibiotalar joints were pricked without injecting cells (sham operation). The therapeutic effects of the hUC-MSCs were evaluated through morphological examination, measurement of hind limb diameter, imaging assessment (X-ray), and measurement of inflammatory factor levels. Spirochete burden was assessed by quantitative real-time polymerase chain reaction (qPCR). Morphological, hind limb diameter, and imaging analyses showed that the low and high hUC-MSC doses significantly reduced bilateral hind limb swelling in the LA mice. Histological (hematoxylin and eosin staining) examination of tibiotalar joint sections showed that when compared with the control group, inflammatory cell infiltration, and bilateral hind limb tissue damage were reduced in the two treatment groups. Enzyme-linked immunosorbent assays revealed that the levels of IL-6 and TNF-α in lysates from the bilateral tibiotarsal joints were significantly lower in the two treatment groups than in the control group. QPCR results showed that hUC-MSCs treatment had no significant effect on the spirochete load in the tibiotarsal joint. Our findings indicate that hUC-MSCs can alleviate inflammation in the KM mouse model of LA without increasing *B. burgdorferi* burden., which is expected to be a new potential method for the treatment of LA.

## 1. Introduction

Lyme disease (LD) is a zoonotic infectious disease caused by the tick-borne spirochete, *Borrelia burgdorferi* (*B. burgdorferi*). LD is common worldwide and has been reported in more than 80 countries, mainly in North Africa, Europe, Asia, North America, and some South American countries [1]. It is estimated that 300,000–400,000 new LD cases are reported annually, with the United States being the most affected country [2]. LD is a complicated multisystem disease, and its clinical manifestations can be divided into three stages. In the first stage, Erythema chronicum migrans is the main manifestation, in the second stage, carditis and neurological disorders occur, and in the third stage, 60% of the patients develop Lyme arthritis (LA) within 2 years [3]. LA, the most common and serious clinical manifestation of advanced LD, is characterized by joint redness, swelling, pain, and effusion, and it mainly affects large joints, for example, knees, with frequent recurrence and lasts for several years [4]. The synovial lesions in LA patients, including synovial thickening, congestion, fibrosis, fibroblast proliferation, and monocyte infiltration, are similar to other forms of chronic arthritis, such as rheumatoid arthritis (RA).

Oral or intravenous antibiotic therapy is beneficial for most patients with LA, which is referred to as antibiotic-responsive LD. Even after *B. burgdorferi* clearance, the host's immune response and inflammation induced by *B. burgdorferi* antigens may persist for a long time. Therefore, 10%–20% of patients with LA receiving standardized antibiotic treatment, synovitis persists for several years or even worsens, and such cases are called antibiotic-refractory LA [5, 6]. Nonsteroidal anti-inflammatory drugs and corticosteroids are usually needed for antibiotic-refractory LA, and if necessary, synovectomy should also be considered [3]. However, because long-term drug therapy has side effects that seriously affect the patients' quality of life, it is necessary to develop safer and effective treatments.

Mesenchymal stem cells (MSCs), which were discovered in the 1970s, are adult stem cells that originate from the mesoderm [7]. These spindle-shaped cells have self-renewal ability and multidirectional differentiation potential, with the capacity to differentiate into osteoblasts, adipocytes, and chondroblasts in vitro [8]. MSCs are abundant and can be easily separated from the umbilical cord, placenta, fat, bone marrow, dental pulp, and menstrual blood [9, 10]. They express various markers, including CD73, CD90, and CD105, but not CD11b, CD19, CD34, CD45, and human leukocyte antigen (HLA)-DR. They have low immunogenicity because they express low levels of HLA-I and rarely express HLA-II [11]. MSCs can differentiate into damaged tissue components and communicate with various immune cells via cell–cell contacts or through paracrine interaction by producing various cytokines, chemokines, growth factors, and extracellular vesicles, thereby, promoting injury repair and immunomodulation [12]. Hence, several preclinical and clinical trials have examined their therapeutic value against various autoimmune and inflammatory diseases, with broad prospects [13]. However, there are no reports about treating LA with MSCs. Therefore, this study involved a preliminary examination of the therapeutic effects of MSCs using a Kunming (KM) mouse model of LA. The most important reason for carrying out this study to explore the role of MSCs in the treatment of LA is to solve the treatment challenges faced by these patients with antibiotic-refractory LA, which is also the original intention and ultimate goal of this study.

## 2. Materials and Methods

### 2.1. Culture of *Borrelia burgdorferi*

*Borrelia burgdorferi* was cultured as described previously [14]. Briefly, the *B. burgdorferi* sensu stricto strain 4680 (DSMZ; catalog number: A1511358-1) was cultured in Barbour–Stoenner–Kelly-II (BSK-II) medium supplemented with 6% rabbit serum (Ruite, Guangzhou, China) at 32–34°C. The spirochetes were then centrifuged at 2500 × *g* for 10 min, washed thrice with sterile phosphate-buffered saline (PBS), and counted using a counting plate under a dark-field microscope. Finally, the spirochetes were resuspended in fresh BSK-II medium at the concentration of 1 × 10^8^ cells/mL for injection into mice.

### 2.2. Mouse Preparation

SPF female KM mice (aged 4–6 weeks) were purchased from the Department of Experimental Zoology, KM Medical University (animal permit number: SCXK [Dian] K 2020-0004). All mice were healthy and were not infected with *B. burgdorferi*. The mice were housed with free access to clean drinking water and food. The study protocol was approved by the Ethical Inspection Committee of Animal Experiment, KM Medical University (approval number: kmmu 20221640). This study adhered to the Guide for the Care and Use of Laboratory Animals [15] and the ARRIVE guidelines for reporting animal research [16]. All animal experiments were conducted by qualified personnel, and relevant regulations were strictly observed to ensure the welfare of experimental animals.

### 2.3. Acquisition and Preparation of Human Umbilical Cord MSCs (hUC-MSCs)

The hUC-MSCs were supplied by Yunnan Health Cell Biotechnology Co., Ltd. The surface markers of MSCs were identified using flow cytometry at the KM KingMed Institute for Clinical Laboratory. The multilineage differentiation potential of hUC-MSCs was examined using multilineage differentiation induction kits (Wuhan Pricella Biotechnology Co., Ltd, Beijing, China; catalog number: PD-017, PD-018 and PD-019) according to the manufacturer's suggested protocols. Then, the ability to develop into adipocytes, osteoblasts, and chondrocytes was evaluated by staining with Oil red O, Alizarin red S and Alisine blue. The hUC-MSCs were cultured using MSC serum-free medium (Yocon Biotechnology Co., Ltd, Beijing, China; catalog number: NC0103) at 37°C (5% CO_2_) and amplified until the fourth to sixth passages before use in subsequent experiments.

### 2.4. Experimental Design and Grouping

**Figure 1:**
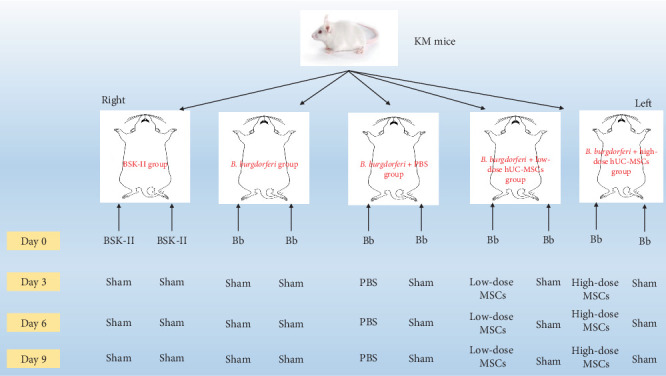
Experimental grouping and design flowchart.

All experiments involving *B. burgdorferi* were conducted in a biosafety level 2 laboratory. After 1 week of adaptive feeding, KM mice were divided into the BSK-II, *B. burgdorferi*, *B. burgdorferi* + PBS, *B. burgdorferi* + low-dose hUC-MSCs (1 × 10^6^ cells per mouse), and *B. burgdorferi* + high-dose hUC-MSCs (2 × 10^6^ cells per mouse) groups and the study was completed within 14 days. On the day 0, 50 μL of sterile BSK-II medium was injected into the bilateral hind foot pads of BSK-II group, while 50 μL of BSK-II medium containing 5 × 10^6^*B. burgdorferi* organisms was injected into the bilateral hind foot pads of other groups. On days 3, 6, and 9, 50 μL of PBS containing 1 × 10^6^ hUC-MSCs and 2 × 10^6^ hUC-MSCs were injected into the right hind foot pads of the *B. burgdorferi* + low-dose and *B. burgdorferi* + high-dose treatment groups, respectively, while the *B. burgdorferi* + PBS group was injected with 50 μL of sterile PBS. The left hind footpads of these three groups above were injected without PBS or hUC-MSCs (sham operation). On days 3, 6, and 9, the bilateral hind footpads of the BSK-II and *B. burgdorferi* groups were injected without PBS or hUC-MSCs (sham operation). To facilitate understanding of the design and grouping of this experiment, we have visualized the process in Figure 1.

### 2.5. Assessment of Swelling Levels in Hind Limbs

The diameter of tibiotalus joint in hind limbs of mice was measured by the same researcher with vernier caliper every day. Vernier calipers were used to determine the length of the diameter when placed in contact with the skin of the mouse joints. On day 13, the hind limb swelling levels of the mice in each group were examined by eye, and the tibiotarsal joints were photographed using a low-dose dental X-ray unit (Tianjie Electronic Co., Ltd, Zhengzhou, China).

### 2.6. Assessment of Pathology

All mice were euthanized on day 13, followed by immediate collection and fixation of tibiotalar joint tissues in 4% paraformaldehyde (Solarbio Life Sciences, Beijing, China) for 48 h at room temperature. They were then embedded in paraffin, sectioned at a thickness of 4 µm, stained with hematoxylin and eosin (Solarbio Life Sciences, Beijing, China) according to manufacturer guidelines, and then examined under a regular microscope.

### 2.7. Assessment of IL-6 and TNF-α Levels in Tibiotalar Joints

Before (day 3), during (day 8), and after (day 13) treatment with hUC-MSCs, a batch of mice was euthanized in each group and tibiotalar joints were collected. The joints were then thoroughly homogenized in RIPA lysis buffer supplemented with protease inhibitors (Solarbio Life Sciences, Beijing, China) and centrifuged at 12,000 × *g* at 4°C, for 10 min. The supernatants were immediately stored at −80°C until they were used for IL-6 and TNF-α concentration analysis using enzyme-linked immunosorbent assay kits (Neobioscience Biotechnology Co., Ltd, Shenzhen, China; catalog numbers: EMC004 and EMC102, respectively) according to the manufacturer's instructions.

### 2.8. Assessment of Spirochete Burden in Tibiotalar Joints

To investigate the impact of MSCs therapy on spirochete load, we humanely euthanized the mice at the experimental end point (day 13). Subsequently, we aseptically collected the bilateral hind limb tibiotarsal joints from the four groups of mice except BSK-II group. Total DNA was extracted using the TIANamp Genomic DNA kit (TIANGEN BIOTECH (BEIJING) Co., Ltd, Beijing, China; catalog numbers: DP304) according to the manufacturer's suggested protocol. We used the methods and primers described in Van Laar's [17] study to detect the copy number of the *B. burgdorferi flaB* gene using quantitative real-time polymerase chain reaction (qPCR), and normalized it with the copy number of mouse β-actin. QPCR was performed using the NovoStart SYBR qPCR SuperMix Plus kit (Novoprotein Scientific Co., Ltd, Suzhou, China; catalog numbers: E096) following the manufacturer's suggested protocol. The spirochete burden was expressed as the number of *B. burgdorferi flaB* copies per 10^4^ mouse β-actin copies.

### 2.9. Statistical Analyses

All data are presented as mean ± standard deviation. Statistical analyses were done on SPSS version 25 (IBM Corporation, Armonk, NY, USA) and data were visualized using GraphPad Prism version 8 (GraphPad Software, San Diego, CA, USA). Hind limb diameter data were analyzed using generalized estimating equations. Differences between the average levels of IL-6, TNF-α, and spirochete burden across the mouse groups were compared using one-way ANOVA. The paired *t*-test was employed to compare the differences in the average levels of IL-6, TNF-α, and spirochete burden between the left and right sides in mice within the same group. *p*  < 0.05 indicated statistically significant differences.

## 3. Results

### 3.1. hUC-MSCs Culture and Identification

**Figure 2:**
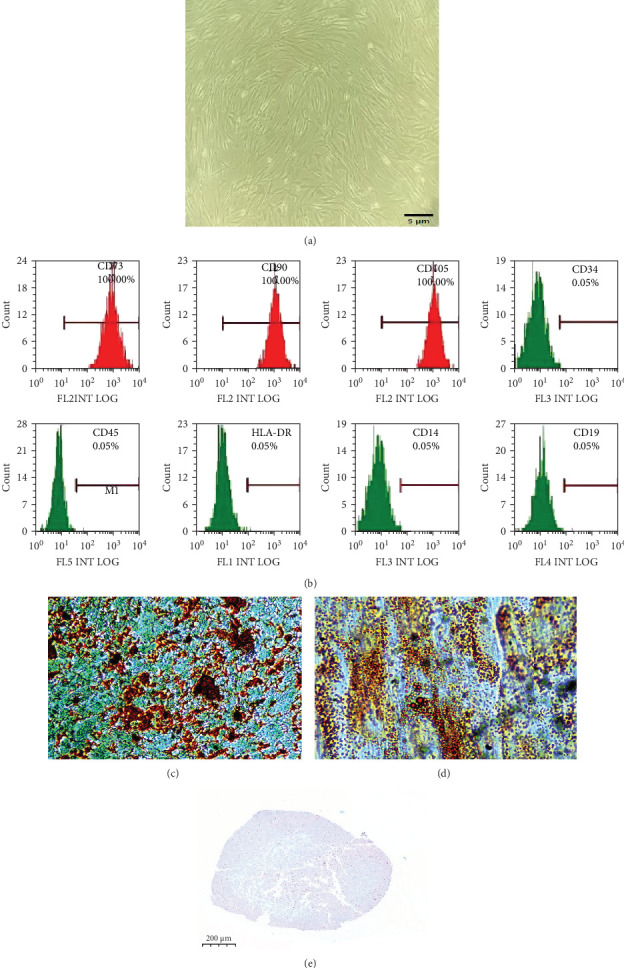
Identification of hUC-MSCs. (a) Cultured hUC-MSCs were examined and imaged under an optical microscope (×100). (b) hUC-MSCs were identified based on indicated surface markers using flow cytometry. (c) Osteogenic differentiation and Alizarin red S staining (×100). (d) Adipogenic differentiation and Oil red O staining (×400). (e) Chondrogenic differentiation and Alixin blue staining (×50). hUC-MSCs, human umbilical cord mesenchymal stem cells.

The hUC-MSCs adhered to the surfaces of the culture dishes and grew well, with a spindle-shaped morphology (Figure 2a). Flow cytometry revealed that the proportion of CD73-, CD90-, and CD105-positive cells was 100%, whereas the proportion of CD19-, CD34-, CD45-, and HLA-DR-positive cells was almost zero (Figure 2b), which is consistent with the abovementioned surface marker characteristics of MSCs. Meanwhile, with the multipotency of hUC-MSCs, we successfully induced them into adipocytes, osteoblasts, and chondroblast, which indicated by Oil red O staining, Alizarin red S staining, and Alixin blue staining, respectively (Figure 2c–e).

### 3.2. hUC-MSCs Therapy Can Effectively Reduce Swelling in Mouse Hind Limbs

**Figure 3:**
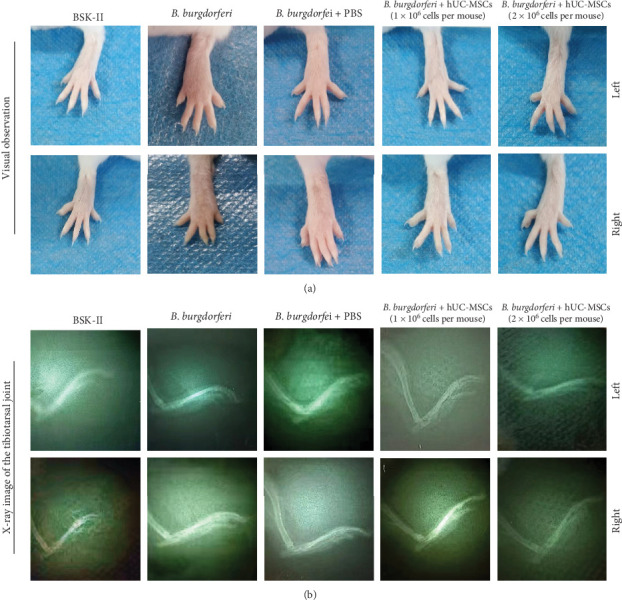
Hind limb swelling in mice from each experimental group was evaluated at the end of the experimental period (day 13). (a) Macroscopic observation. (b) Images of the tibiotarsal joints using a low-dose dental X-ray unit. In both treatment groups, swelling was markedly reduced in both hind limbs. BSK-II, Barbour–Stoenner–Kelly-II medium; *B. burgdorferi*, *Borrelia burgdorferi*; hUC-MSCs, human umbilical cord mesenchymal stem cells.

At the end of the experimental period (day 13), our analyses revealed that when compared with *B. burgdorferi* and *B. burgdorferi* + PBS groups, the low- and high-dose hUC-MSC-treated groups had significantly lower bilateral hind limb swelling (Figure 3a). X-ray imaging also showed that in the two treatment groups, edema in the soft tissues around the tibiotalar joints was significantly reduced compared with *B. burgdorferi* and *B. burgdorferi* + PBS groups (Figure 3b).

**Figure 4:**
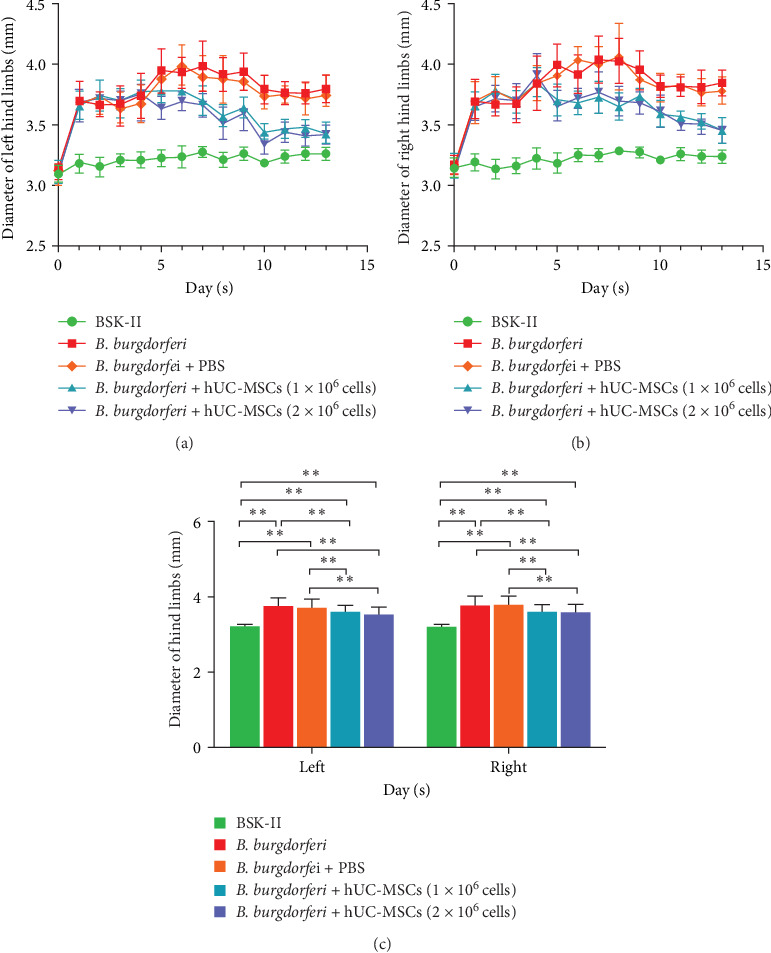
Comparison of the hind limb diameters of mice from different groups. (a) The trend of left hind limb diameter changes in each group over time. (b) The trend of right hind limb diameter changes in each group over time. (c) Comparison of the overall hind limb diameter in each mouse group. Treatment with hUC-MSCs significantly reduced bilateral hind limb diameters, which did not differ significantly between the two treatment groups. Analyses of whether differences were statistically significant were conducted using generalized estimating equations. *⁣*^*∗*^ and *⁣*^*∗∗*^ indicate *p*  < 0.05 and  < 0.01, respectively. *n* = 11 in BSK-II group, *n* = 16 per group (in the other four groups). BSK-II, Barbour–Stoenner–Kelly-II medium; *B. burgdorferi*, *Borrelia burgdorferi*; hUC-MSCs, human umbilical cord mesenchymal stem cells.

Assessment of mouse hind limb diameter found that in the two treatment groups, the bilateral hind limb diameters began to decrease on the fifth day and were significantly lower than those recorded in the *B. burgdorferi* and *B. burgdorferi* + PBS groups until the end of the experiment period (Figure 4a,b). However, they did not differ between the two treatment groups (Figure 4c).

### 3.3. In the Two Treatment Groups and the *B. burgdorferi* + PBS Group, the Diameter of the Left Hind Limb Was Lower Than the Right Side of the Same Group

**Figure 5:**
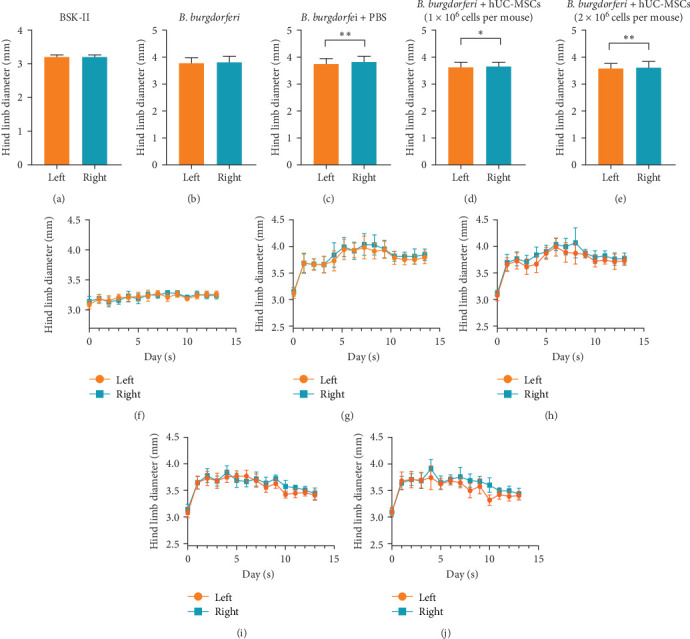
Comparison of the left and right hind limb diameters within each mouse group. (a–e) Overall comparison of the left and right hind limb diameters within the same group. (f–j) The trend of bilateral hind limb diameter changes in each group over time. In the two treatment groups and the *B. burgdorferi* + PBS group, the right hind limb diameters were significantly higher than the left hind limb diameters in the same groups. Analyses of whether differences were statistically significant were conducted using generalized estimating equations. *⁣*^*∗*^ and *⁣*^*∗∗*^ indicate *p*  < 0.05 and  < 0.01, respectively. *n* = 11 in BSK-II group, *n* = 16 per group (in the other four groups). BSK-II, Barbour–Stoenner–Kelly-II medium; *B. burgdorferi*, *Borrelia burgdorferi*; hUC-MSCs, human umbilical cord mesenchymal stem cells.

The left hind limb diameters of mice in the BSK-II and *B. burgdorferi* groups were not significantly different when compared with the right hind limbs in the same groups (Figure 5a,b,f,g). However, the right hind limb diameters of the *B. burgdorferi* + PBS group and the two treatment groups were significantly higher than the left hind limb diameters in the same groups (Figure 5c–e,h–j). The average diameter of the left hind limb in the *B. burgdorferi* + PBS group, *B. burgdorferi* + low-dose hUC-MSCs group, and *B. burgdorferi* + high-dose hUC-MSCs group was 3.73 ± 0.0161, 3.59 ± 0.0121, and 3.55 ± 0.0132 mm, respectively, while the right side was 3.81 ± 0.0135, 3.63 ± 0.0122, and 3.62 ± 0.0109 mm, respectively.

### 3.4. Treatment With hUC-MSCs Improves Mouse Tibiotarsal Joint Pathological Changes

**Figure 6:**
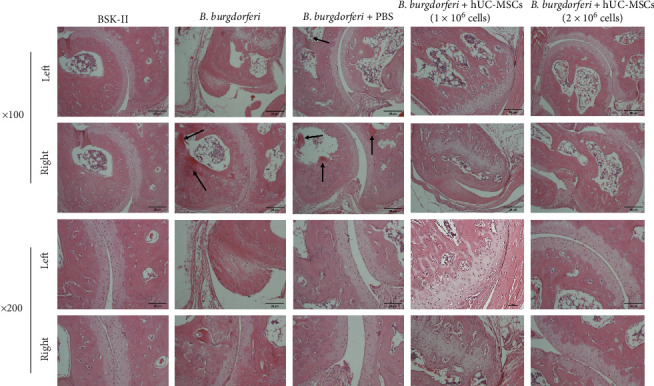
Comparison of tibiotarsal joint histopathological changes in each group. Images of representative hematoxylin and eosin-stained tibiotarsal joint sections are shown. The tibiotarsus tissue structures of mice from the *B. burgdorferi*; and *B. burgdorferi* + PBS groups were disordered and they exhibited more inflammatory cell infiltration and some necrotic niduses (indicated by the arrows). These pathological changes were improved in the two treatment groups, especially in the high-dose hUC-MSCs treatment group in which the tissue structure recovered to the normal condition. BSK-II, Barbour–Stoenner–Kelly-II medium; *B. burgdorferi*, *Borrelia burgdorferi*; hUC-MSCs; human umbilical cord mesenchymal stem cells.

Histological analysis revealed a disordered tibiotarsus structure in mice from the *B. burgdorferi* and *B. burgdorferi* + PBS groups, which exhibited more inflammatory cell infiltration and some necrotic niduses (indicated by the arrows) compared with the other three groups. However, treatment with hUC-MSCs improved these pathological changes dose-dependently, with a marked improvement in the high-dose hUC-MSCs treatment group, which recovered to the same degree as the BSK-II group and exhibited a close-to-normal tissue structure, without edema, necrosis, and exudation (Figure 6).

### 3.5. Treatment With hUC-MSCs Significantly Reduced the Levels of IL-6 and TNF-α in the Tibiotarsus

**Figure 7:**
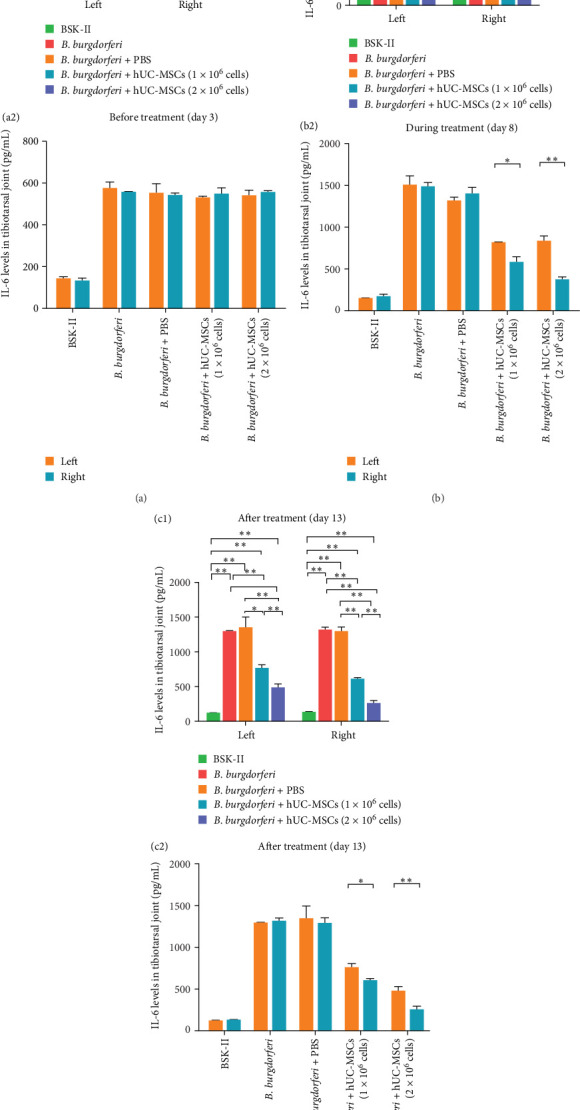
(a–c) Comparison of IL-6 levels in the tibiotarsal joints of mice from each group before, during, and after treatment. (a1–c1) Comparisons between groups. The levels of IL-6 in the bilateral tibiotalar joints of mice from the two treatment groups were significantly lower during and after treatment. After treatment, IL-6 levels were lower in the left and right sides of the high-dose-treated group than in the low-dose-treated group. (a2–c2) Comparisons of the IL-6 levels in the left and right sides within the same groups. During and after treatment, the IL-6 levels on the right sides of both treatment groups were lower than on the left sides of the same groups. Differences between the different groups were compared using one-way ANOVA. The differences between the left and right sides within the same group were assessed using a paired t-test for comparison. *⁣*^*∗*^ and *⁣*^*∗∗*^ indicate *p*  < 0.05 and  < 0.01, respectively. *n* = 3 per group on day3 and day 8. *n* = 4 per group on day13. BSK-II, Barbour–Stoenner–Kelly-II medium; *B. burgdorferi*, *Borrelia burgdorferi*; hUC-MSCs; human umbilical cord mesenchymal stem cells.

Before treatment with hUC-MSCs (day 3), the levels of IL-6 and TNF-α in the bilateral tibiotarsuses of the four *B. burgdorferi*-inoculated groups were significantly higher than in the BSK-II group (Figures 7a1 and 8a1), and there were no significant differences between the left and right tibiotarsal joints in all groups (Figure 7a2 and 8a2).

**Figure 8:**
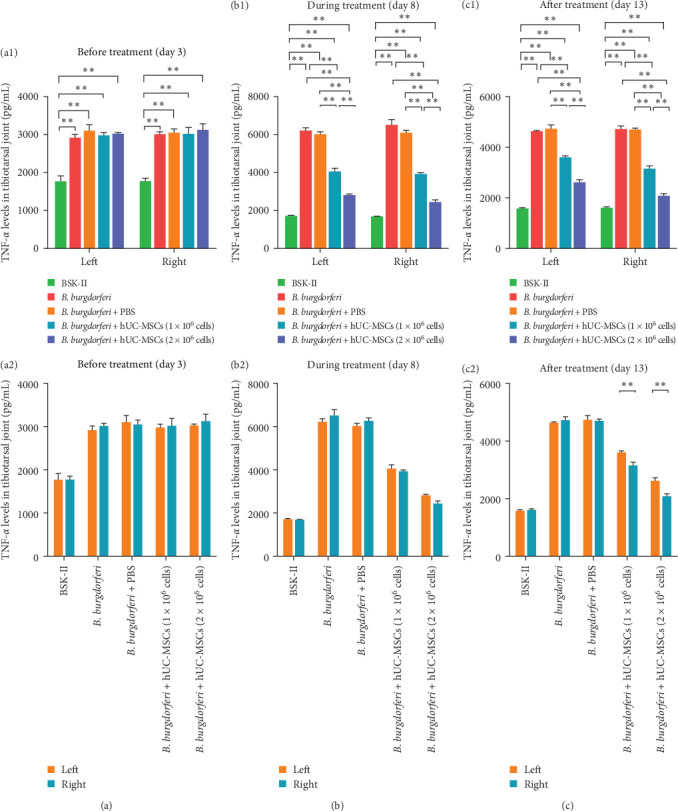
(a–c) Comparison of TNF-α levels in the tibiotarsal joints of mice from each group before, during, and after treatment. (a1–c1) Comparisons between groups. The levels of TNF-α in the bilateral tibiotalar joints of mice from the two treatment groups were significantly lower during and after treatment, and TNF-α levels in the left or right sides were lower in the high-dose-treated group than in the low-dose-treated group. (a2–c2) Comparisons of TNF-α levels in the left and right sides within the same groups. After treatment, TNF-α levels on the right sides of both treatment groups were lower than those on the left sides of the same groups. Differences between the different groups were compared using one-way ANOVA. The differences between the left and right sides within the same group were assessed using a paired t-test for comparison. *⁣*^*∗*^ and *⁣*^*∗∗*^ indicate *p*  < 0.05 and  < 0.01, respectively. n=3 per group on day3 and day 8. n=4 per group on day13. BSK-II, Barbour–Stoenner–Kelly-II medium; *B. burgdorferi*, *Borrelia burgdorferi*; hUC-MSCs, human umbilical cord mesenchymal stem cells.

During the treatment period (day 8), when compared with the *B. burgdorferi* and *B. burgdorferi* + PBS groups, the IL-6 levels in the bilateral tibiotarsuses of the two treatment groups were significantly lower. However, although the levels on the left sides of the two treatment groups were not different, they were significantly different on the right side (Figure 7b1). A comparison of IL-6 levels on the left and right sides within each of the two treatment groups revealed that they were significantly lower on the right side than on the left side (Figure 7b2). When compared with *B. burgdorferi* and *B. burgdorferi* + PBS groups, the levels of TNF-α were significantly lower on both sides of the two treatment groups. Moreover, when compared with the low-dose hUC-MSCs-treated group, the levels of TNF-α were significantly lower on the left and right sides in the high-dose hUC-MSCs-treated group (Figure 8b1). A comparison of the left and right sides of the two treatment groups (within the same group) revealed no significant differences in TNF-α levels (Figure 8b2).

After the treatment (at the end of the experiment, day 13), the bilateral levels of IL-6 and TNF-α in the two treatment groups were significantly lower than in the *B. burgdorferi* and *B. burgdorferi* + PBS groups. Moreover, when compared with the low-dose hUC-MSCs-treated group, their levels on the left and right sides were significantly lower in the high-dose hUC-MSCs-treated group (Figures 7c1 and 8c1). A comparison of the left and right sides of the two treatment groups (within the same group) revealed that the levels of IL-6 and TNF-α were significantly lower on the right side than on the left side (Figures 7c2 and 8c2).

### 3.6. MSCs Treatment Has No Significant Effect on the Spirochete Load in the Mice Tibiotarsal Joints

**Figure 9:**
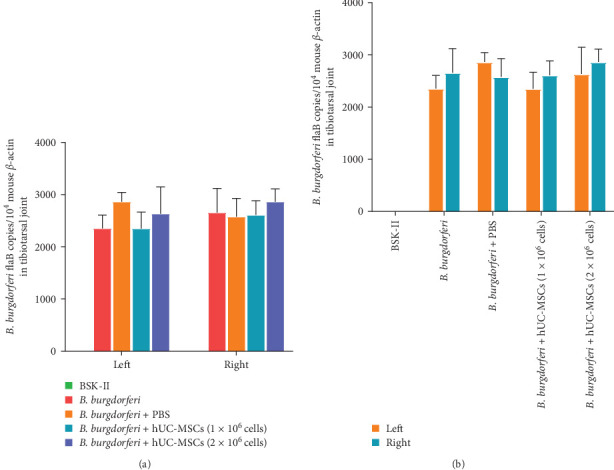
Comparison of spirochete burden in the tibiotarsal joints of mice. (a) Comparisons between different groups. (b) Comparisons of spirochete burden in the left and right sides within the same groups. No statistically significant differences were observed, neither between different groups on the same side nor between the left and right sides within the same group. Differences between the different groups were compared using one-way ANOVA. The differences between the left and right sides within the same group were assessed using a paired *t*-test for comparison. *n* = 4 per group. BSK-II, Barbour–Stoenner–Kelly-II medium; *B. burgdorferi*, *Borrelia burgdorferi*; hUC-MSCs, human umbilical cord mesenchymal stem cells.

QPCR results showed that the spirochete burden in the tibiotarsal joints of the two MSCs-treated groups of mice was not significantly different from that of the *B. burgdorferi* and *B. burgdorferi* + PBS groups, while the BS K-II group, being uninfected with *B. burgdorferi*, had a burden of zero (Figure 9a). Within the same group, there are no significant differences between the left and right sides (Figure 9b).

## 4. Discussion

The pathogenesis of LA is not completely clear. It is generally believed that the symptoms associated with LD are mainly caused by the host's immune response to *B. burgdorferi*, rather than the toxins produced by *B. burgdorferi* [18]. The surface of *B. burgdorferi* is rich in lipoproteins, such as the outer surface proteins (Osps), which have strong antigenicity and are the main trigger of host immune responses. The main Osps are OspA, OspB, and OspC [19].

LD triggers strong innate and adaptive immune responses. After *B. burgdorferi* infection, its surface lipoprotein activate pattern recognition receptors, such as Toll-like receptor (TLR)-1, TLR-2, TLR-4, and TLR-5, which induces an inflammatory reaction and activates innate immunity, thereby, contributing to the clearance of *B. burgdorferi*. However, uncontrolled inflammation aggravates the disease [20, 21]. In addition, *B. burgdorferi* components like DNA and RNA, which are released after its phagocytosis and degradation, can also activate the TLR-8 and TLR-9 signaling pathways [22], which activate NF-κB signaling, drive the production of inflammatory cytokines, chemokines, and type I interferons, and initiate an anti-infective immune response to *B. burgdorferi*. The adaptive immunity mediated by Th1 cells, B cells, and their antibodies, and Th17 cells also participate in LA pathogenesis [4, 23].

After antibiotic treatment, a small number of patients with LA have severe synovitis, which is called anti-inflammatory LA or postinfectious LA. The synovial lesions of these patients are characterized by high levels of synovial fibroblast proliferation, monocyte–macrophage infiltration, marked vascular proliferation, and in some patients, occluded microvascular injury and microscopic bleeding. These pathological changes are very similar to those of RA and other autoimmune diseases [24]. Postinfectious LA is associated with both spirochaeta and host factors. Infection with the highly inflammatory strain of *B. burgdorferi*, RST1, commonly results in postinfectious LA [25]. The continued activation of immune response and inflammation by *B. burgdorferi* residues is also a factor in postinfectious LA. Peptidoglycan, a major component of the cell wall of *B. burgdorferi* that is shed during replication is extremely difficult to clear and may remain in the synovial fluid for years after antibiotic treatment [26]. More importantly, long-term excessive and uncontrolled host inflammatory responses, as well as the lack of anti-inflammatory cytokines, result in a failure to downregulate inflammation and restore homeostasis after the spirochaeta has been killed, ultimately leading to vascular damage and autoimmune response. For example, transcriptomic analysis of synovial samples from patients with postinfectious LA revealed a high enrichment of genes associated with innate and adaptive immune responses and antigen presentation [27], and a deficiency of the classic anti-inflammatory cytokine, IL-10, during postinfectious LA, is strong evidence [23]. Because MSCs can exert immunosuppression through cell–cell contact, downregulation of antigen-presenting and costimulatory molecules, and secretion of anti-inflammatory cytokines like IL-10, they have the potential to treat various autoimmune diseases [28], such as RA. Several in vitro, preclinical and clinical studies have investigated the treatment of RA with MSCs, with most clinical studies revealing that they have good therapeutic effects and safety [29]. For example, a large-scale nonrandomized controlled trial treated 36 patients with RA using the conventional drug treatment and 136 patients with intravenously injected UC-MSCs (4 × 10^7^) combined with the conventional drug treatment. This study showed that treatment with UC-MSCs significantly reduced disease activity and significantly alleviated clinical symptoms when compared with the control group. The study also found that UC-MSCs significantly increased the levels of IL-4 secretion by Th2 cells, increased the percentage of CD4^+^CD25^+^FoxP3^+^ Treg cells in the peripheral blood, and decreased the levels of TNF-α and IL-6 [30]. Another phase I/II prospective study with a follow-up period of up to 3 years also showed that treatment with UC-MSCs can significantly improve symptoms and reduce various proinflammatory factors in RA patients and that their efficacy can be maintained for up to 3 years [31]. However, there are no reports on the use of MSCs to treat LA, especially antibiotic-refractory LA, which has an RA-like pathogenesis. Based on LA pathogenesis and the immunomodulatory effects of MSCs, we conducted a preliminary study in mice.

In this study, KM mice were used to establish an LA model, in which viable *B. burgdorferi* in BSK-II medium was inoculated into the bilateral hind limbs, followed by treatment with different doses of hUC-MSCs in PBS, which were inoculated into the right hind limbs of the mice. PBS was used as the negative control, whereas the left hind limbs were injected without drugs (sham operation). Our analyses revealed that in the two treatment groups, the degree of swelling on the right side, in which hUC-MSCs were directly transplanted, was significantly lower when compared with the control group. Moreover, the tibiotarsal joint pathological changes induced by *B. burgdorferi* injection were improved and the levels of the inflammatory cytokines, IL-6 and TNF-α, were significantly reduced. Although the left side received the sham operation only, the abovementioned changes were also observed on the left sides in the two treatment groups. We also compared the left and right sides in the two treatment groups with the expectation that the effect on the left side, which was not directly inoculated with hUC-MSCs, would be inferior or at most, equivalent to that observed on the right side. As expected, within the same treatment groups, compared with the left side, the reduction of IL-6/TNF-α levels on the right side (the side directly treated with MSCs) was more obvious. However, we observed that the diameter on the left side was smaller than on the right side. We hypothesize that this phenomenon was caused by the injection procedure on the right side. In the two treatment groups and the *B. burgdorferi* + PBS group, 50 μLs of liquid were injected into the right hind limb. This injection volume interfered with the diameter measurement results, which partially masked the therapeutic effect of the hUC-MSCs. Based on the interesting experimental results we have discovered, and given that the hUC-MSCs used in our study do not differentiate into mouse tissue components to achieve a repair effect, we hypothesize that the therapeutic effect of hUC-MSCs may be achieved through paracrine effects, where numerous substances are secreted into the bloodstream, thereby, exerting a global immunoregulation effect, or through migration of the stem cells into the ipsilateral joint and exerting local immunoregulation, or a combination of both. However, we emphasize that the current data are not enough to fully illustrate the specific mechanisms. The mechanism proposed is only our hypotheses and speculations, which may provide some clues for future research. Notably, the effects of the high-dose hUC-MSC treatment on IL-6 and TNF-α levels, as well as the pathological changes, were superior to those of the low-dose treatment, although hind limb diameter did not differ between the two groups. We speculate that this phenomenon occurred because the therapeutic effect of the high-dose hUC-MSCs may be better than that of the low-dose, but such dose effects may only be reflected at the micro level, and not at the macro level. Increasing the hUC-MSC dose may cause the difference to be reflected at the macro level.

Inflammation, as a protective response of the body to injury and pathogens, has a double-edged sword effect on the body [32]. We should also realize its beneficial side in removing pathogens. Although the above results show that MSCs can alleviate inflammation and improve symptoms in LA mouse models, will it promote the growth of spirochetes in vivo while playing an immunomodulatory role? To clarify this issue, we assessed the spirochete burden in the tibiotarsal joints of different groups of mice. Fortunately, our results show that MSCs treatment has no significant effect on spirochete load, which also suggests that MSCs alleviate inflammation without disrupting the body's natural process of spirochete clearance.

This study has some limitations. First, because of experimental condition limitations, we only tested two hUC-MSCs doses, which were inoculated locally, and we did not use MSCs from other tissues. Second, the study used only one mouse strain. Hence, to better demonstrate the feasibility and efficacy of MSCs in the treatment of LA, future studies should consider expanding the hUC-MSC dose gradient, using multistrain mice, and testing MSCs from different tissues.

## 5. Conclusion

This study shows that in LA mice, hUC-MSCs can significantly reduce hind limb swelling, improve tibiotarsal joint pathological changes, and reduce the levels of the inflammatory cytokines, IL-6 and TNF-α. Our findings indicate that hUC-MSCs can reduce inflammation in the KM mouse model of LA without increasing *B. burgdorferi* burden, which is expected to be a new potential method for the treatment of LA. The specific mechanisms of therapeutic effects are not yet fully understood and require further research to be explored.

## Data Availability

All data generated or analyzed during this study are included in this published article.

## Nomenclature

LD: Lyme disease
*B. burgdorferi*: *Borrelia burgdorferi*
LA: Lyme arthritis
RA: Rheumatoid arthritis
MSCs: Mesenchymal stem cells
HLA: Human leukocyte antigen
KM: Kunming
hUC-MSCs: Human umbilical cord mesenchymal stem cells
BSK-II medium: Barbour–Stoenner–Kelly-II (BSK-II) medium
PBS: Phosphate-buffered saline
Osps: Outer surface proteins
TLR: Toll-like receptor.
